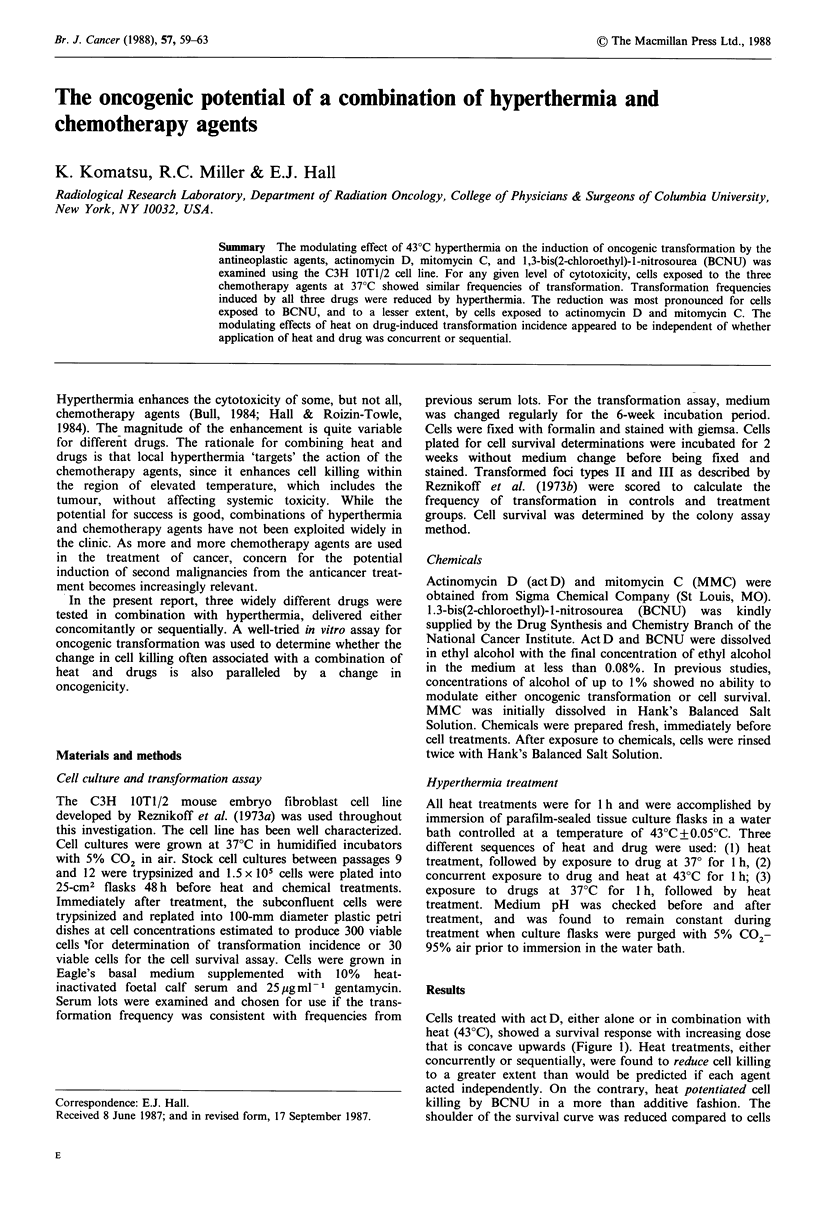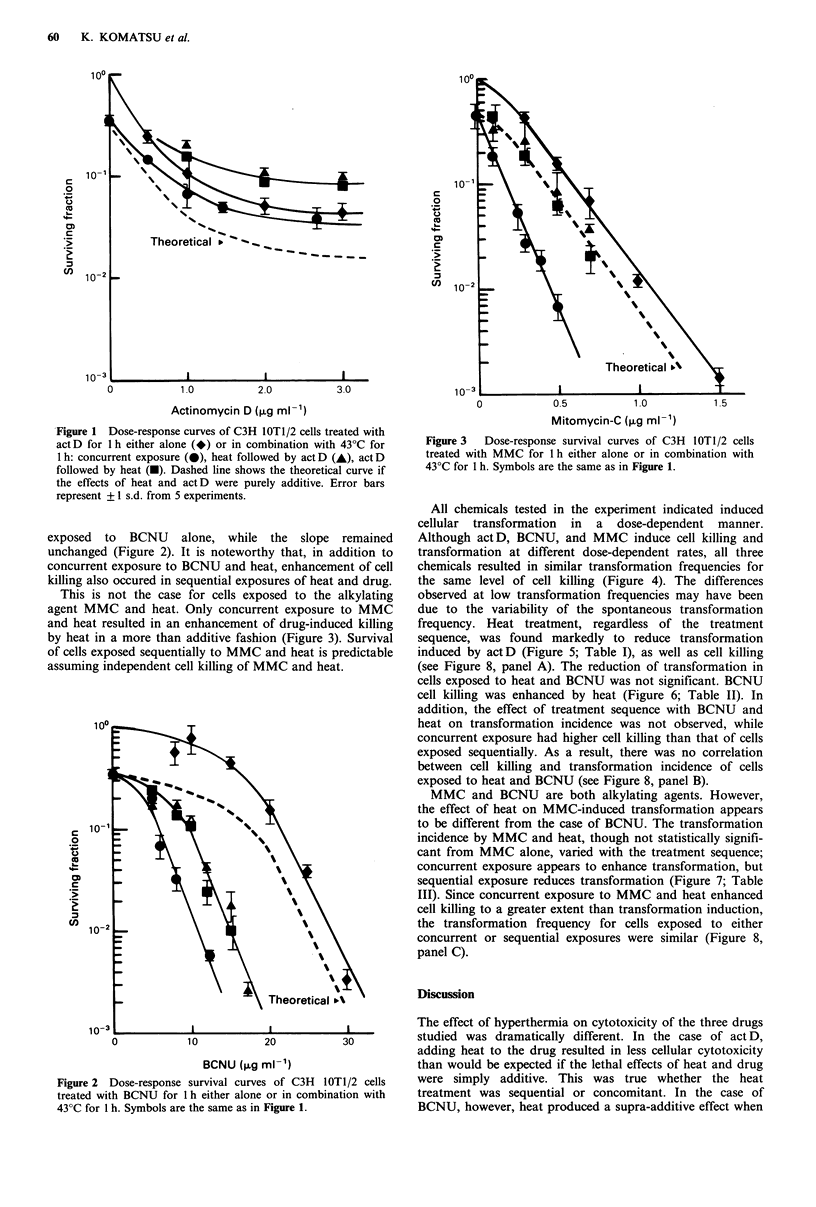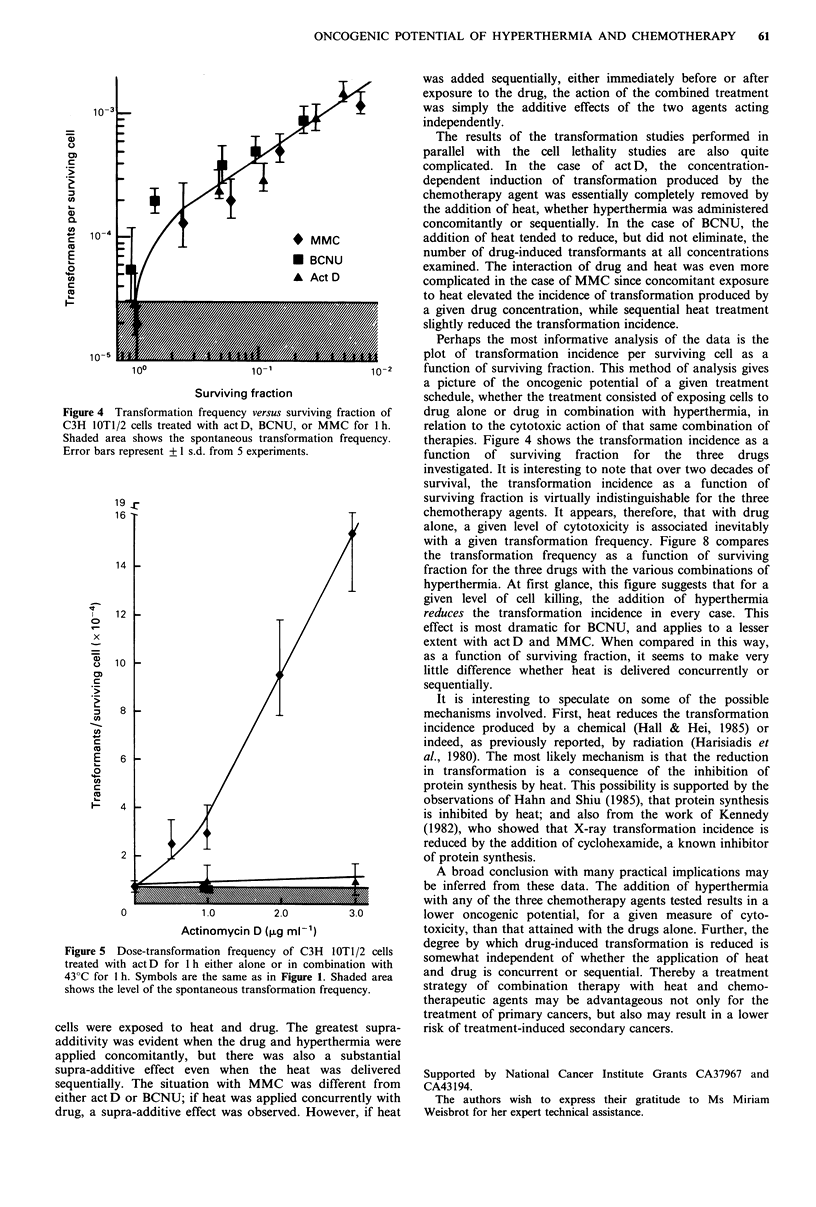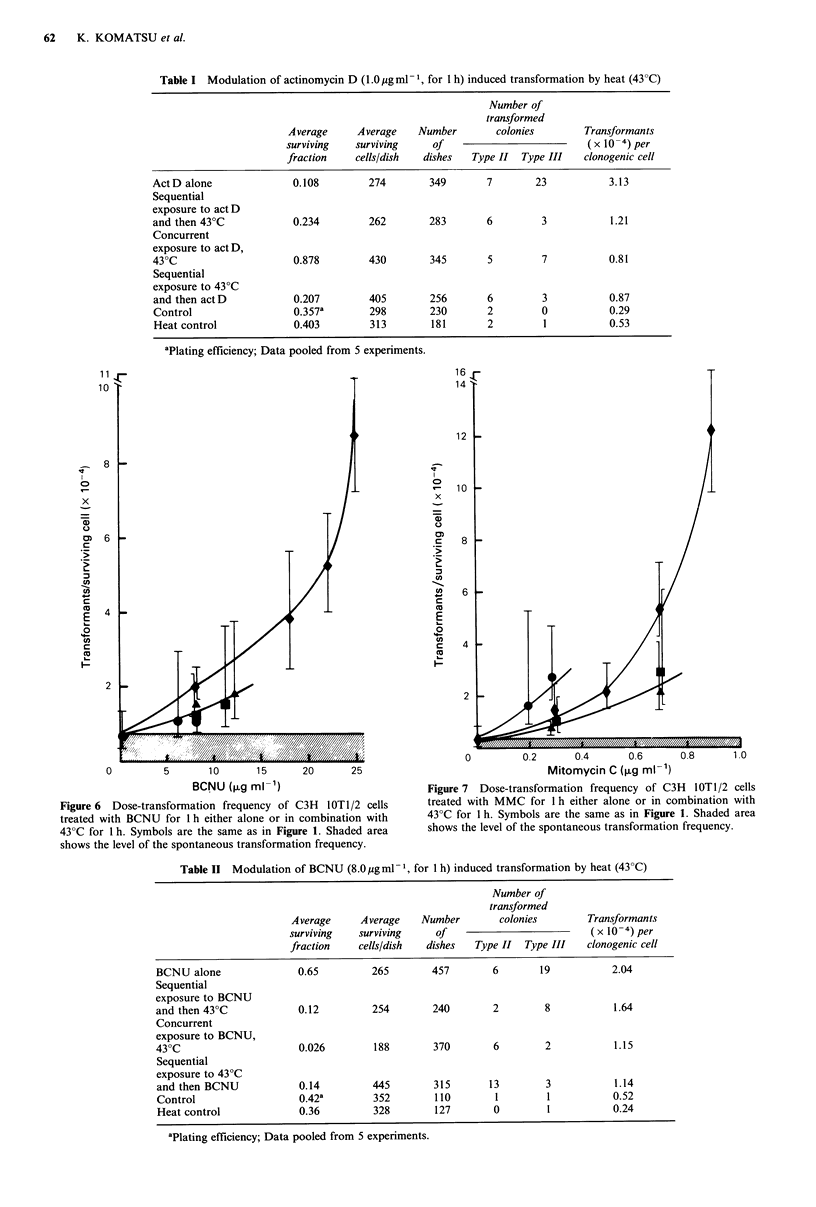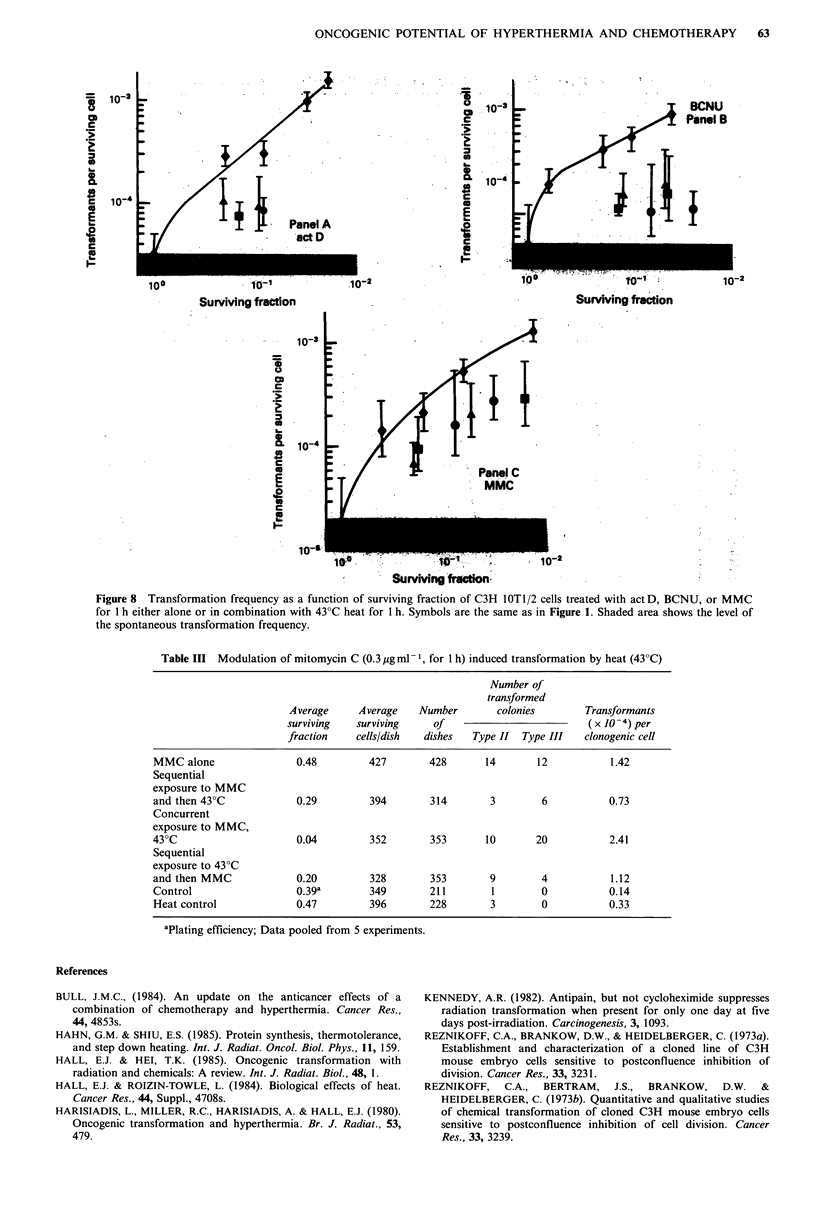# The oncogenic potential of a combination of hyperthermia and chemotherapy agents.

**DOI:** 10.1038/bjc.1988.9

**Published:** 1988-01

**Authors:** K. Komatsu, R. C. Miller, E. J. Hall

**Affiliations:** Department of Radiation Oncology, College of Physicians & Surgeons of Columbia University, New York, NY 10032.

## Abstract

The modulating effect of 43 degrees C hyperthermia on the induction of oncogenic transformation by the antineoplastic agents, actinomycin D, mitomycin C, and 1,3-bis(2-chloroethyl)-1-nitrosourea (BCNU) was examined using the C3H 10T1/2 cell line. For any given level of cytotoxicity, cells exposed to the three chemotherapy agents at 37 degrees C showed similar frequencies of transformation. Transformation frequencies induced by all three drugs were reduced by hyperthermia. The reduction was most pronounced for cells exposed to BCNU, and to a lesser extent, by cells exposed to actinomycin D and mitomycin C. The modulating effects of heat on drug-induced transformation incidence appeared to be independent of whether application of heat and drug was concurrent or sequential.


*Br. J. Cancer* (1988), **57**, 59–63 © The Macmillan Press Ltd., 1988

### The oncogenic potential of a combination of hyperthermia and chemotherapy agents

K. Komatsu, R.C. Miller & E.J. Hall

*Radiological Research Laboratory, Department of Radiation Oncology, College of Physicians & Surgeons of Columbia University, New York, NY 10032, USA.*

**Summary** The modulating effect of 43°C hyperthermia on the induction of oncogenic transformation by the antineoplastic agents, actinomycin D, mitomycin C, and 1,3-bis(2-chloroethyl)-1-nitrosourea (BCNU) was examined using the C3H 10T1/2 cell line. For any given level of cytotoxicity, cells exposed to the three chemotherapy agents at 37°C showed similar frequencies of transformation. Transformation frequencies induced by all three drugs were reduced by hyperthermia. The reduction was most pronounced for cells exposed to BCNU, and to a lesser extent, by cells exposed to actinomycin D and mitomycin C. The modulating effects of heat on drug-induced transformation incidence appeared to be independent of whether application of heat and drug was concurrent or sequential.

Hyperthermia enhances the cytotoxicity of some, but not all, chemotherapy agents (Bull, 1984; Hall & Roizin-Towle, 1984). The magnitude of the enhancement is quite variable for different drugs. The rationale for combining heat and drugs is that local hyperthermia 'targets' the action of the chemotherapy agents, since it enhances cell killing within the region of elevated temperature, which includes the tumour, without affecting systemic toxicity. While the potential for success is good, combinations of hyperthermia and chemotherapy agents have not been exploited widely in the clinic. As more and more chemotherapy agents are used in the treatment of cancer, concern for the potential induction of second malignancies from the anticancer treatment becomes increasingly relevant.

In the present report, three widely different drugs were tested in combination with hyperthermia, delivered either concomitantly or sequentially. A well-tried *in vitro* assay for oncogenic transformation was used to determine whether the change in cell killing often associated with a combination of heat and drugs is also paralleled by a change in oncogenicity.

### Materials and methods

### *Cell culture and transformation assay*

The C3H 10T1/2 mouse embryo fibroblast cell line developed by Reznikoff *et al.* (1973*a*) was used throughout this investigation. The cell line has been well characterized. Cell cultures were grown at 37°C in humidified incubators with 5% $CO_2$ in air. Stock cell cultures between passages 9 and 12 were trypsinized and $1.5 \times 10^5$ cells were plated into 25-cm$^2$ flasks 48 h before heat and chemical treatments. Immediately after treatment, the subconfluent cells were trypsinized and replated into 100-mm diameter plastic petri dishes at cell concentrations estimated to produce 300 viable cells for determination of transformation incidence or 30 viable cells for the cell survival assay. Cells were grown in Eagle's basal medium supplemented with 10% heat-inactivated foetal calf serum and 25 $\mu$g ml$^{-1}$ gentamycin. Serum lots were examined and chosen for use if the transformation frequency was consistent with frequencies from previous serum lots. For the transformation assay, medium was changed regularly for the 6-week incubation period. Cells were fixed with formalin and stained with giemsa. Cells plated for cell survival determinations were incubated for 2 weeks without medium change before being fixed and stained. Transformed foci types II and III as described by Reznikoff *et al.* (1973*b*) were scored to calculate the frequency of transformation in controls and treatment groups. Cell survival was determined by the colony assay method.

### *Chemicals*

Actinomycin D (act D) and mitomycin C (MMC) were obtained from Sigma Chemical Company (St Louis, MO). 1.3-bis(2-chloroethyl)-1-nitrosourea (BCNU) was kindly supplied by the Drug Synthesis and Chemistry Branch of the National Cancer Institute. Act D and BCNU were dissolved in ethyl alcohol with the final concentration of ethyl alcohol in the medium at less than 0.08%. In previous studies, concentrations of alcohol of up to 1% showed no ability to modulate either oncogenic transformation or cell survival. MMC was initially dissolved in Hank's Balanced Salt Solution. Chemicals were prepared fresh, immediately before cell treatments. After exposure to chemicals, cells were rinsed twice with Hank's Balanced Salt Solution.

### *Hyperthermia treatment*

All heat treatments were for 1 h and were accomplished by immersion of parafilm-sealed tissue culture flasks in a water bath controlled at a temperature of 43°C$\pm$0.05°C. Three different sequences of heat and drug were used: (1) heat treatment, followed by exposure to drug at 37° for 1 h, (2) concurrent exposure to drug and heat at 43°C for 1 h; (3) exposure to drugs at 37°C for 1 h, followed by heat treatment. Medium pH was checked before and after treatment, and was found to remain constant during treatment when culture flasks were purged with 5% $CO_2$–95% air prior to immersion in the water bath.

### Results

Cells treated with act D, either alone or in combination with heat (43°C), showed a survival response with increasing dose that is concave upwards (Figure 1). Heat treatments, either concurrently or sequentially, were found to *reduce* cell killing to a greater extent than would be predicted if each agent acted independently. On the contrary, heat *potentiated* cell killing by BCNU in a more than additive fashion. The shoulder of the survival curve was reduced compared to cells

Correspondence: E.J. Hall.
Received 8 June 1987; and in revised form, 17 September 1987.

**Figure 1** Dose-response curves of C3H 10T1/2 cells treated with act D for 1 h either alone (◆) or in combination with 43°C for 1 h: concurrent exposure (●), heat followed by act D (▲), act D followed by heat (■). Dashed line shows the theoretical curve if the effects of heat and act D were purely additive. Error bars represent ±1 s.d. from 5 experiments.

exposed to BCNU alone, while the slope remained unchanged (Figure 2). It is noteworthy that, in addition to concurrent exposure to BCNU and heat, enhancement of cell killing also occured in sequential exposures of heat and drug.

This is not the case for cells exposed to the alkylating agent MMC and heat. Only concurrent exposure to MMC and heat resulted in an enhancement of drug-induced killing by heat in a more than additive fashion (Figure 3). Survival of cells exposed sequentially to MMC and heat is predictable assuming independent cell killing of MMC and heat.

**Figure 2** Dose-response survival curves of C3H 10T1/2 cells treated with BCNU for 1 h either alone or in combination with 43°C for 1 h. Symbols are the same as in **Figure 1**.

**Figure 3** Dose-response survival curves of C3H 10T1/2 cells treated with MMC for 1 h either alone or in combination with 43°C for 1 h. Symbols are the same as in **Figure 1**.

All chemicals tested in the experiment indicated induced cellular transformation in a dose-dependent manner. Although act D, BCNU, and MMC induce cell killing and transformation at different dose-dependent rates, all three chemicals resulted in similar transformation frequencies for the same level of cell killing (Figure 4). The differences observed at low transformation frequencies may have been due to the variability of the spontaneous transformation frequency. Heat treatment, regardless of the treatment sequence, was found markedly to reduce transformation induced by act D (Figure 5; Table I), as well as cell killing (see Figure 8, panel A). The reduction of transformation in cells exposed to heat and BCNU was not significant. BCNU cell killing was enhanced by heat (Figure 6; Table II). In addition, the effect of treatment sequence with BCNU and heat on transformation incidence was not observed, while concurrent exposure had higher cell killing than that of cells exposed sequentially. As a result, there was no correlation between cell killing and transformation incidence of cells exposed to heat and BCNU (see Figure 8, panel B).

MMC and BCNU are both alkylating agents. However, the effect of heat on MMC-induced transformation appears to be different from the case of BCNU. The transformation incidence by MMC and heat, though not statistically significant from MMC alone, varied with the treatment sequence; concurrent exposure appears to enhance transformation, but sequential exposure reduces transformation (Figure 7; Table III). Since concurrent exposure to MMC and heat enhanced cell killing to a greater extent than transformation induction, the transformation frequency for cells exposed to either concurrent or sequential exposures were similar (Figure 8, panel C).

### Discussion

The effect of hyperthermia on cytotoxicity of the three drugs studied was dramatically different. In the case of act D, adding heat to the drug resulted in less cellular cytotoxicity than would be expected if the lethal effects of heat and drug were simply additive. This was true whether the heat treatment was sequential or concomitant. In the case of BCNU, however, heat produced a supra-additive effect when

**Figure 4** Transformation frequency *versus* surviving fraction of C3H 10T1/2 cells treated with act D, BCNU, or MMC for 1 h. Shaded area shows the spontaneous transformation frequency. Error bars represent $\pm 1$ s.d. from 5 experiments.

**Figure 5** Dose-transformation frequency of C3H 10T1/2 cells treated with act D for 1 h either alone or in combination with 43°C for 1 h. Symbols are the same as in **Figure 1**. Shaded area shows the level of the spontaneous transformation frequency.

cells were exposed to heat and drug. The greatest supra-additivity was evident when the drug and hyperthermia were applied concomitantly, but there was also a substantial supra-additive effect even when the heat was delivered sequentially. The situation with MMC was different from either act D or BCNU; if heat was applied concurrently with drug, a supra-additive effect was observed. However, if heat was added sequentially, either immediately before or after exposure to the drug, the action of the combined treatment was simply the additive effects of the two agents acting independently.

The results of the transformation studies performed in parallel with the cell lethality studies are also quite complicated. In the case of act D, the concentration-dependent induction of transformation produced by the chemotherapy agent was essentially completely removed by the addition of heat, whether hyperthermia was administered concomitantly or sequentially. In the case of BCNU, the addition of heat tended to reduce, but did not eliminate, the number of drug-induced transformants at all concentrations examined. The interaction of drug and heat was even more complicated in the case of MMC since concomitant exposure to heat elevated the incidence of transformation produced by a given drug concentration, while sequential heat treatment slightly reduced the transformation incidence.

Perhaps the most informative analysis of the data is the plot of transformation incidence per surviving cell as a function of surviving fraction. This method of analysis gives a picture of the oncogenic potential of a given treatment schedule, whether the treatment consisted of exposing cells to drug alone or drug in combination with hyperthermia, in relation to the cytotoxic action of that same combination of therapies. Figure 4 shows the transformation incidence as a function of surviving fraction for the three drugs investigated. It is interesting to note that over two decades of survival, the transformation incidence as a function of surviving fraction is virtually indistinguishable for the three chemotherapy agents. It appears, therefore, that with drug alone, a given level of cytotoxicity is associated inevitably with a given transformation frequency. Figure 8 compares the transformation frequency as a function of surviving fraction for the three drugs with the various combinations of hyperthermia. At first glance, this figure suggests that for a given level of cell killing, the addition of hyperthermia *reduces* the transformation incidence in every case. This effect is most dramatic for BCNU, and applies to a lesser extent with act D and MMC. When compared in this way, as a function of surviving fraction, it seems to make very little difference whether heat is delivered concurrently or sequentially.

It is interesting to speculate on some of the possible mechanisms involved. First, heat reduces the transformation incidence produced by a chemical (Hall & Hei, 1985) or indeed, as previously reported, by radiation (Harisiadis *et al.*, 1980). The most likely mechanism is that the reduction in transformation is a consequence of the inhibition of protein synthesis by heat. This possibility is supported by the observations of Hahn and Shiu (1985), that protein synthesis is inhibited by heat; and also from the work of Kennedy (1982), who showed that X-ray transformation incidence is reduced by the addition of cyclohexamide, a known inhibitor of protein synthesis.

A broad conclusion with many practical implications may be inferred from these data. The addition of hyperthermia with any of the three chemotherapy agents tested results in a lower oncogenic potential, for a given measure of cytotoxicity, than that attained with the drugs alone. Further, the degree by which drug-induced transformation is reduced is somewhat independent of whether the application of heat and drug is concurrent or sequential. Thereby a treatment strategy of combination therapy with heat and chemotherapeutic agents may be advantageous not only for the treatment of primary cancers, but also may result in a lower risk of treatment-induced secondary cancers.

Supported by National Cancer Institute Grants CA37967 and CA43194.

The authors wish to express their gratitude to Ms Miriam Weisbrot for her expert technical assistance.

**Table I** Modulation of actinomycin D (1.0 μg ml$^{-1}$, for 1 h) induced transformation by heat (43°C)

| | *Average surviving fraction* | *Average surviving cells/dish* | *Number of dishes* | *Number of transformed colonies* | | *Transformants (× 10$^{-4}$) per clonogenic cell* |
|---|---|---|---|---|---|---|
| | | | | *Type II* | *Type III* | |
| Act D alone | 0.108 | 274 | 349 | 7 | 23 | 3.13 |
| Sequential exposure to act D and then 43°C | 0.234 | 262 | 283 | 6 | 3 | 1.21 |
| Concurrent exposure to act D, 43°C | 0.878 | 430 | 345 | 5 | 7 | 0.81 |
| Sequential exposure to 43°C and then act D | 0.207 | 405 | 256 | 6 | 3 | 0.87 |
| Control | 0.357[a] | 298 | 230 | 2 | 0 | 0.29 |
| Heat control | 0.403 | 313 | 181 | 2 | 1 | 0.53 |

[a]Plating efficiency; Data pooled from 5 experiments.

**Figure 6** Dose-transformation frequency of C3H 10T1/2 cells treated with BCNU for 1 h either alone or in combination with 43°C for 1 h. Symbols are the same as in **Figure 1**. Shaded area shows the level of the spontaneous transformation frequency.

**Figure 7** Dose-transformation frequency of C3H 10T1/2 cells treated with MMC for 1 h either alone or in combination with 43°C for 1 h. Symbols are the same as in **Figure 1**. Shaded area shows the level of the spontaneous transformation frequency.

**Table II** Modulation of BCNU (8.0 μg ml$^{-1}$, for 1 h) induced transformation by heat (43°C)

| | *Average surviving fraction* | *Average surviving cells/dish* | *Number of dishes* | *Number of transformed colonies* | | *Transformants (× 10$^{-4}$) per clonogenic cell* |
|---|---|---|---|---|---|---|
| | | | | *Type II* | *Type III* | |
| BCNU alone | 0.65 | 265 | 457 | 6 | 19 | 2.04 |
| Sequential exposure to BCNU and then 43°C | 0.12 | 254 | 240 | 2 | 8 | 1.64 |
| Concurrent exposure to BCNU, 43°C | 0.026 | 188 | 370 | 6 | 2 | 1.15 |
| Sequential exposure to 43°C and then BCNU | 0.14 | 445 | 315 | 13 | 3 | 1.14 |
| Control | 0.42[a] | 352 | 110 | 1 | 1 | 0.52 |
| Heat control | 0.36 | 328 | 127 | 0 | 1 | 0.24 |

[a]Plating efficiency; Data pooled from 5 experiments.

**Figure 8** Transformation frequency as a function of surviving fraction of C3H 10T1/2 cells treated with act D, BCNU, or MMC for 1 h either alone or in combination with 43°C heat for 1 h. Symbols are the same as in **Figure 1**. Shaded area shows the level of the spontaneous transformation frequency.

**Table III** Modulation of mitomycin C (0.3 $\mu g\,ml^{-1}$, for 1 h) induced transformation by heat (43°C)

| | *Average surviving fraction* | *Average surviving cells/dish* | *Number of dishes* | *Number of transformed colonies* | | *Transformants ($\times 10^{-4}$) per clonogenic cell* |
|---|---|---|---|---|---|---|
| | | | | *Type II* | *Type III* | |
| MMC alone | 0.48 | 427 | 428 | 14 | 12 | 1.42 |
| Sequential exposure to MMC and then 43°C | 0.29 | 394 | 314 | 3 | 6 | 0.73 |
| Concurrent exposure to MMC, 43°C | 0.04 | 352 | 353 | 10 | 20 | 2.41 |
| Sequential exposure to 43°C and then MMC | 0.20 | 328 | 353 | 9 | 4 | 1.12 |
| Control | 0.39[a] | 349 | 211 | 1 | 0 | 0.14 |
| Heat control | 0.47 | 396 | 228 | 3 | 0 | 0.33 |

[a]Plating efficiency; Data pooled from 5 experiments.

### References

BULL, J.M.C., (1984). An update on the anticancer effects of a combination of chemotherapy and hyperthermia. *Cancer Res.*, **44**, 4853s.

HAHN, G.M. & SHIU, E.S. (1985). Protein synthesis, thermotolerance, and step down heating. *Int. J. Radiat. Oncol. Biol. Phys.*, **11**, 159.

HALL, E.J. & HEI, T.K. (1985). Oncogenic transformation with radiation and chemicals: A review. *Int. J. Radiat. Biol.*, **48**, 1.

HALL, E.J. & ROIZIN-TOWLE, L. (1984). Biological effects of heat. *Cancer Res.*, **44**, Suppl., 4708s.

HARISIADIS, L., MILLER, R.C., HARISIADIS, A. & HALL, E.J. (1980). Oncogenic transformation and hyperthermia. *Br. J. Radiat.*, **53**, 479.

KENNEDY, A.R. (1982). Antipain, but not cycloheximide suppresses radiation transformation when present for only one day at five days post-irradiation. *Carcinogenesis*, **3**, 1093.

REZNIKOFF, C.A., BRANKOW, D.W., & HEIDELBERGER, C. (1973*a*). Establishment and characterization of a cloned line of C3H mouse embryo cells sensitive to postconfluence inhibition of division. *Cancer Res.*, **33**, 3231.

REZNIKOFF, C.A., BERTRAM, J.S., BRANKOW, D.W. & HEIDELBERGER, C. (1973*b*). Quantitative and qualitative studies of chemical transformation of cloned C3H mouse embryo cells sensitive to postconfluence inhibition of cell division. *Cancer Res.*, **33**, 3239.